# Clinical relevance of pulse pressure variations for predicting fluid responsiveness in mechanically ventilated intensive care unit patients: the grey zone approach

**DOI:** 10.1186/s13054-014-0587-9

**Published:** 2014-11-04

**Authors:** Matthieu Biais, Stephan Ehrmann, Arnaud Mari, Benjamin Conte, Yazine Mahjoub, Olivier Desebbe, Julien Pottecher, Karim Lakhal, Dalila Benzekri-Lefevre, Nicolas Molinari, Thierry Boulain, Jean-Yves Lefrant, Laurent Muller, with the collaboration of AzuRea Group

**Affiliations:** Département des Urgences, Hôpital Pellegrin, CHU de Bordeaux, F-33076 Bordeaux Cedex, France and University Bordeaux Segalen, Bordeaux, France; Service de Réanimation Polyvalente, CHRU de Tours, 2 boulevard Tonnellé, F37044 Tours cedex 9, France; Hôpitaux universitaires de Toulouse, Département d’Anesthésie-Réanimation, 31059 Toulouse, France et Université Paul Sabatier, Equipe d’Accueil 4564, Toulouse, France; Service des Réanimations, Division Anesthésie, Réanimation, Urgences, Douleur, CHU Nîmes, Place du Professeur Robert Debré, 30029 Nîmes Cedex 9, France; Unité de réanimation polyvalente, CHU Amiens, Amiens, France; Hospices Civils de Lyon, Groupement Hospitalier Est, Department of Anesthesiology and Intensive Care, Louis Pradel Hospital, Claude Bernard Lyon 1 University, Lyon, France; Réanimation Chirurgicale, Service dAnesthésie-Réanimation Chirurgicale, Pôle Anesthésie-Réanimations Chirurgicales-SAMU-SMUR, Hôpital de Hautepierre, Hôpitaux Universitaires de Strasbourg, 1 Avenue Molière, 67098 Strasbourg Cedex, France; Réanimation Chirurgicale Polyvalente, Service d’Anesthésie-Réanimation, Hôpital Nord Laennec, Centre Hospitalier Universitaire de Nantes, Boulevard Jacques Monod, Saint Herblain, 44093 Nantes cedex 1, France; Service de Réanimation Médicale, Hôpital La Source, Centre Hospitalier Régional, avenue de l’Hôpital, 45067 Orléans Cedex 1, France; Department of Statistics, University of Montpellier Lapeyronie Hospital, UMR 729 MISTEA, Montpellier, France

## Abstract

**Introduction:**

Pulse pressure variation (PPV) has been shown to predict fluid responsiveness in ventilated intensive care unit (ICU) patients. The present study was aimed at assessing the diagnostic accuracy of PPV for prediction of fluid responsiveness by using the grey zone approach in a large population.

**Methods:**

The study pooled data of 556 patients from nine French ICUs. Hemodynamic (PPV, central venous pressure (CVP) and cardiac output) and ventilator variables were recorded. Responders were defined as patients increasing their stroke volume more than or equal to 15% after fluid challenge. The receiver operating characteristic (ROC) curve and grey zone were defined for PPV. The grey zone was evaluated according to the risk of fluid infusion in hypoxemic patients.

**Results:**

Fluid challenge led to increased stroke volume more than or equal to 15% in 267 patients (48%). The areas under the ROC curve of PPV and CVP were 0.73 (95% confidence interval (CI): 0.68 to 0.77) and 0.64 (95% CI 0.59 to 0.70), respectively (*P* <0.001). A grey zone of 4 to 17% (62% of patients) was found for PPV. A tidal volume more than or equal to 8 ml.kg^−1^ and a driving pressure (plateau pressure - PEEP) more than 20 cmH_2_O significantly improved the area under the ROC curve for PPV. When taking into account the risk of fluid infusion, the grey zone for PPV was 2 to 13%.

**Conclusions:**

In ventilated ICU patients, PPV values between 4 and 17%, encountered in 62% patients exhibiting validity prerequisites, did not predict fluid responsiveness.

## Introduction

In intensive care units (ICUs), a fluid challenge based on clinical criteria leads to a significant increase in cardiac output (CO) in approximately 50% of patients [1]. Improving ability to predict fluid responsiveness is of particular interest, given that both persistent hypovolemia and fluid overload are associated with poor clinical outcomes [2-7]. In mechanically ventilated ICU patients, dynamic variables such as pulse pressure variation (PPV) have been shown to be more accurate in predicting fluid responsiveness than static variables such as central venous pressure (CVP) [1,8]. The principle of predicting fluid responsiveness through PPV is based on the transmission of positive respiratory pressure generated by controlled mechanical ventilation to the intra-thoracic vascular compartment [9]. Therefore, spontaneous breathing, mechanical ventilation with low tidal volume (V_T_ <8 ml/kg^−1^), low plateau pressure, low pulmonary compliance and low heart rate to respiratory rate (HR/RR) ratio reduce the accuracy of PPV in predicting fluid responsiveness [10-15]. These findings challenge the idea that a low PPV value (that is <13%) excludes fluid responsiveness, whereas a high PPV value accurately predicts fluid responsiveness (as defined by a 15% increase in CO after fluid infusion). A recent study suggests that PPV values <6% could be associated with fluid responsiveness whereas PPV values ≥10% were highly predictive of a positive response to fluid challenge [10]. The overlap of PPV values between responders and non-responders reported in previous studies [10,11,15] could be interpreted as a ‘grey zone’ in which a clinical decision cannot be made with sufficient certainty [16-19]. The grey zone methodology avoids the binary response proposed by the receiver operating characteristic (ROC) curve methodology, which does not take into account the existence of an overlap between responders and non-responders [16-19]. In contrast, the grey zone approach proposes a low cut-off value that excludes fluid responsiveness in 90% of patients (favouring negative predictive value), whereas a high cut-off value predicts fluid responsiveness in 90% of cases (favouring positive predictive value) [20]. Between these two cut-off values, no decision can actually be taken. Using this method in the anaesthetic setting, Cannesson *et al*. [18] demonstrated that the grey zone approach identifies a range of PPV values between 9% and 13% for which fluid responsiveness could not be reliably predicted. However, ventilator settings for ICU patients and for patients ventilated during general anaesthesia are different. In this previous report by Cannesson *et al*. [18], the V_T_ was 7.9 ± 1.3 ml/kg body weight, with 51% of patients being ventilated with V_T_ ≥8 ml/kg^−1^. We recently reported that during anaesthesia, patients were mechanically ventilated with a mean V_T_ =8.8 ± 1.4 ml/kg^−1^ ideal body weight (IBW) and 18% patients were ventilated with a V_T_ >10 ml/kg^−1^ IBW [21]. In ICU patients, a low V_T_ (<8 ml/kg^−1^) has been shown to improve patient outcome in acute respiratory distress syndrome (ARDS) patients and to prevent the occurrence of acute lung injury in mechanically ventilated patients [22-25]. Moreover, our group recently reported that the use of a low V_T_ associated with positive expiratory pressure and recruitment manoeuvres decreases postoperative complication rate and length of stay in ICU [26]. Therefore, applying the grey zone approach could be informative of the real value of PPV for predicting fluid responsiveness in ICU patients ventilated with low V_T_.

The present study was aimed at assessing the diagnostic accuracy of PPV for prediction of fluid responsiveness by using the grey zone approach in a large ICU population. As a secondary endpoint, we studied respiratory variables that could affect the accuracy of PPV to predict fluid responsiveness (that is V_T_, respiratory driving pressure, respiratory system compliance and HR/RR). We also defined a model for benefit-risk assessment of fluid administration to further evaluate the predictive value of PPV in patients according to their partial arterial oxygen pressure (P_a_O_2_) to inspiratory oxygen fraction (F_i_O_2_) ratio.

## Methods

The present study pooled data obtained from eight published studies (460 patients) in nine French hospitals (one multicentre study having involved ICUs of Orleans, Tours and Paris Bichat hospital) [10,27-32]. The different local Institutional Review Boards gave their approval for performing previous published studies. In addition, some unpublished data for 96 patients were added. As the physician in charge of the patient prescribed a fluid challenge as part of routine care, the Institutional Review Board of Nîmes, France gave its approval (CHU Nîmes Interface Recherche Bioéthique, IRB number 12-03-03) to perform the present study in new patients who were not included in previous published studies. The patient and/or his/her authorized representative were systematically informed and could decline participation.

### Inclusion/exclusion criteria

Mechanically ventilated and sedated patients with acute circulatory failure in whom a fluid challenge was indicated participated in the study. Acute circulatory failure was defined as systolic arterial blood pressure <90 mmHg or mean arterial pressure <65 mmHg with signs of hypoperfusion (oliguria less than 0.5 ml.kg^−1^/h^−1^, arterial lactate >2.5 mMol/L^−1^, presence of skin mottling, unsuccessful attempt to decrease vasopressor infusion rate). Patients with spontaneous breathing, cardiac arrhythmias, unsatisfactory cardiac echogenicity (in patients in whom CO was assessed by echography), increase in intra-abdominal pressure suspected by clinical context and examination, known tricuspid insufficiency, or cardiogenic pulmonary oedema were excluded. Moribund, parturient patients and those younger than 18 years were not included.

### Studied variables

For each patient, age, sex, weight, height, body mass index (BMI), and Acute Physiology and Chronic Health Evaluation (APACHE) II score [33] or the Simplified Acute Physiology Score II (SAPS II) score [34] were recorded at admission. IBW (kg) was defined as follows: X +0.91(height (cm) - 152.4), (X = 50 for men and 45.5 for women). Before performing the fluid challenge, the aetiology of acute circulatory failure and the infusion rates of inotropic and/or vasopressor agents were recorded. For ventilator variables, V_T_ (ml/kg^−1^ of IBW), RR (cycles/min^−1^), the level of positive end-expiratory pressure (PEEP, cmH_2_O) and plateau pressure (cmH_2_O), F_i_O_2_, and P_a_O_2_ (mmHg) were recorded when available. The following haemodynamic variables were recorded: HR (beats.min^−1^/bpm) and mean arterial blood pressure (MAP, mmHg). MAP and CVP (when available) were measured invasively with a zero referenced to the middle axillary line. PPV was calculated as follows [8]:

$$ \mathrm{P}\mathrm{P}\mathrm{V}\ \left(\%\right) = 100\ \mathrm{X}\ 2\left[\left(\mathrm{PPmax}\ \hbox{-}\ \mathrm{P}\mathrm{P} \min \right)/\left(\mathrm{PPmax} + \mathrm{P}\mathrm{P} \min \right)\right]. $$

(PP: pulsed pressure)

In each measurement and after verifying the absence of cardiac arrhythmia and spontaneous breathing, CO was measured either by thermodilution technique (PiCCO system (Pulsion, Medical Systems AG, Munich, Germany) or pulmonary artery catheter (CO-set system (Edwards Lifesciences, Irvine, CA, USA)), echocardiography (General Electric Vivid3 machine; GE Healthcare, Chalfont St. Giles, Buckinghamshire, UK or Acuson CV-70; Siemens Medical, Germany) or oesophageal Doppler (HemoSonic 100; Arrow International, Everett, MA, USA).

### Intervention and definition of fluid responsiveness

When indicated, a fluid challenge using either colloid or isotonic crystalloid solutions was performed over 15 to 30 minutes. The infused volume was most often 500 ml (500 ml in 527 patients and 20 mL/IBW^−1^ in 29 patients, as performed by Reuter *et al*. [35]). Fluid responsiveness was defined as an increase in stroke volume (SV) ≥15% compared to baseline value. Studied variables were measured immediately prior to and two to five minutes following the fluid challenge. When the fluid challenge was repeated, only the first was included in the analysis. Ventilatory settings and inotropic and/or vasopressors drug regimens were kept constant as set by the attending physician during the study period.

### Statistical analysis

We expected to include a large sample of patients with at least 100 events (responders) to allow an accurate determination of the ROC curves and cut-off values. Assuming a proportion of responders close to 50%, about 200 patients would be necessary. However, because we intended to perform bootstrap analysis and subgroup analyses, we decided to include at least 500 patients, a number close to that previously used by Cannesson *et al*. [18].

Results are expressed according to variable distributions (mean ± standard deviation (SD) or median (95% confidence interval (95% CI)) for quantitative variables and frequencies with percentages for qualitative variables. Patients were divided into responders and non-responders according to response to fluid challenge. Comparisons were performed using unpaired Student *t* tests, Mann-Whitney tests, chi-square test, and the Fisher exact method when appropriate. Receiver operating characteristic (ROC) curves were created to assess the discriminative power of PPV and CVP to predict the effect of fluid challenge. The ROC curves were also created by using a bootstrap methodology, which creates multiple samples (1,000) by randomly drawing instances, with replacement, from the original study population [36]. Bootstrapping has been previously used for assigning measures of accuracy to sample estimates and the sample distribution [18,36]. This method limits the impact of outliers and provides more robust representations. The area under the ROC curves with 95% CI was calculated. The comparison of two areas under the ROC curves was performed as previously described by DeLong *et al*. [37].

### Threshold and grey zone determination

The grey zone approach determines a range of values for which no conclusion may be drawn concerning potential fluid responsiveness [16-19,38]. The best threshold for a ROC curve was defined as that which maximizes the Youden index (sensitivity + specificity −1) [17]. A two-step procedure was performed in order to determine the grey zone. The first step consisted in determining the best threshold in each of the 1,000 bootstrapped populations for PPV and CVP. The 95% CI of the best threshold was defined by the observed distributions of the thresholds in the 1,000 populations [36]. The second step was aimed at determining the values for which no conclusive information could be provided concerning fluid responsiveness. We defined inconclusive responses for values with sensitivity <90% or specificity <90% (diagnostic tolerance of 10%). Two-curve (sensitivity, specificity) representation was provided to illustrate this second step. The grey zone was defined as the values that did not allow a 10% diagnostic tolerance. Nevertheless, if the characteristics of the study population produce a 95% CI of the best thresholds larger than the inconclusive zone, the values obtained during the first step were retained as the grey zone. This two-step procedure allows us to provide robust results not impacted by potential outliers. This approach is particularly interesting when small samples (or rare endpoints) are considered. Because there is no clear consensus for statistical comparison between two grey zones, the percentages of patients in the grey zone for PPV and CVP were compared.

Moreover, grey zones were determined according to potential factors that could impact on the ability of PPV to predict fluid responsiveness.

In patients with ARDS, restrictive fluid management has been shown to be associated with a higher number of ventilator-free days at Day_28_ (ARDS network) and a lower mortality rate [39-41]. In patients with ARDS, the value of P_a_O_2_/F_i_O_2_ ratio could influence the clinician’s decision as to perform a fluid challenge or not. For example, a P_a_O_2_/F_i_O_2_ < 100 with impaired left ventricular function could influence the clinician towards avoiding unnecessary volume loading. In contrast, a patient with a P_a_O_2_/F_i_O_2_ ratio ≥200 and a preserved/normal left ventricular function is a patient in whom fluid loading may be beneficial and the risk of fluid overload may be low. Therefore, a cost ratio (R = cost (false positive)/cost (false negative)) was defined according to the recent international definition of ARDS [42]. When the P_a_O_2_/F_i_O_2_ ratio was <100 (severe ARDS group), R was arbitrarily defined as 2 (potential risk = fluid overload). When the P_a_O_2_/F_i_O_2_ ratio was <200 (moderate ARDS group), R was defined as 1 and when the P_a_O_2_/F_i_O_2_ ratio was ≥200 (mild ARDS group), R was defined as 0.5 (potential risk = hypovolemia). All *P* values were two-sided, and a *P* value <0.05 was considered significant. Statistical analyses were performed with R 2.15.1 (The R Foundation for Statistical Computing, Vienna, Austria).

## Results

**Figure 1:**
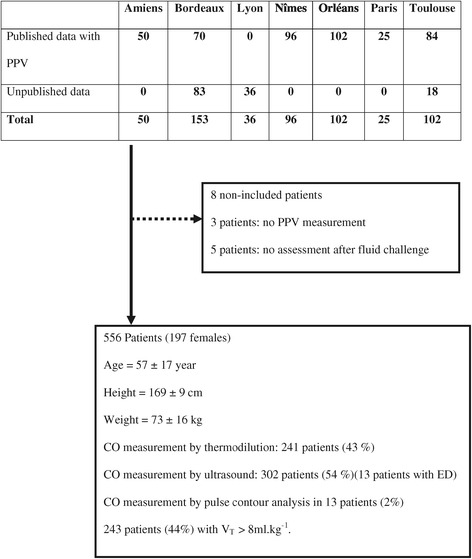
**Flow chart of the study.**

**Figure 2:**
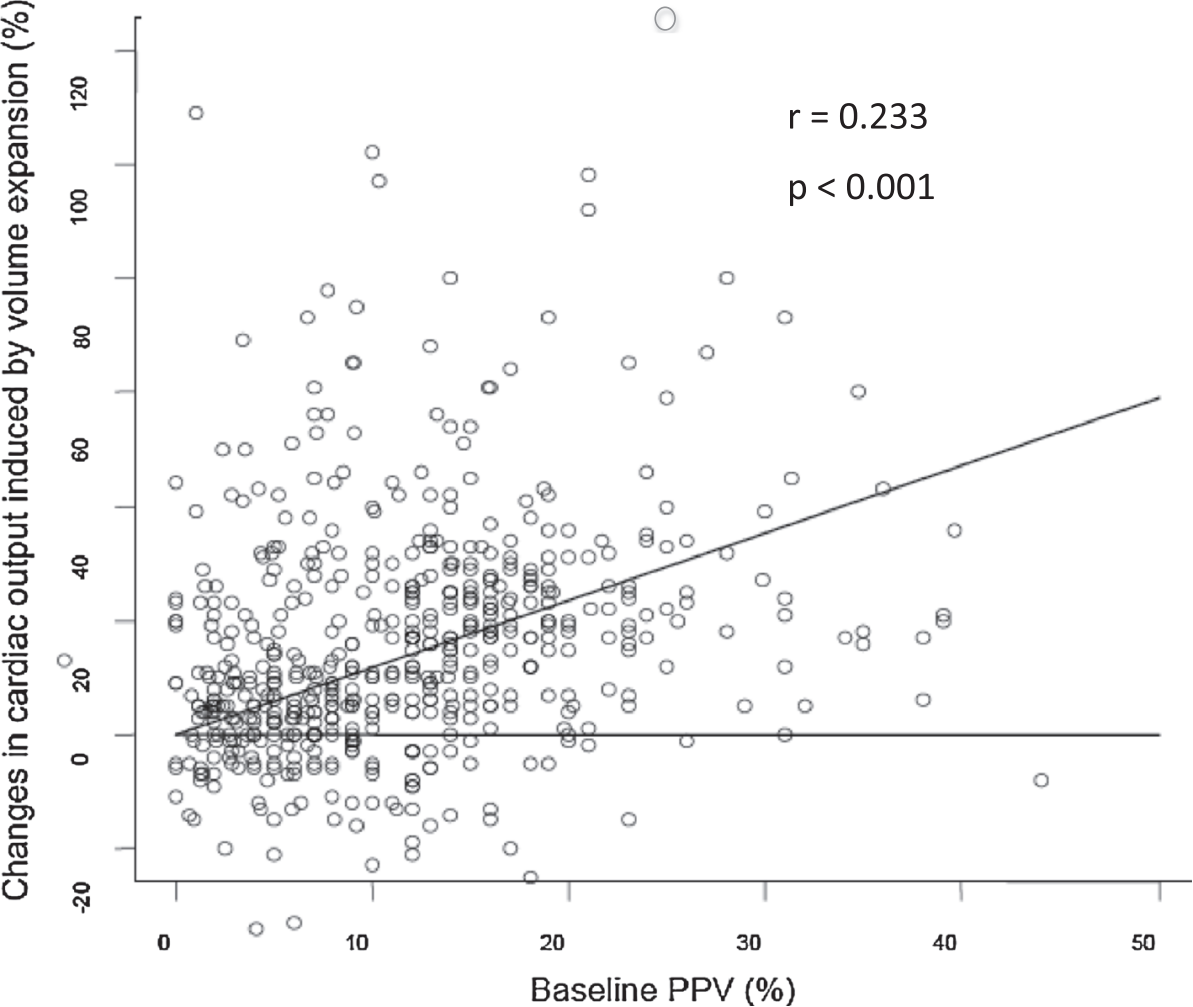
**Changes in cardiac output (%) induced by volume expansion according to the baseline PPV value (%).** PPV, pulse pressure variation.

Data from 564 ICU patients were collected (Figure 1). The assessment of PPV was missing in three patients and no fluid responsiveness was assessed in five other patients. Therefore, 556 patients (197 women) were analysed (Figure 1). Most of the CO measurements were obtained through thermodilution or ultrasound technique (Figure 1). Two hundred and forty-three patients (44%) were ventilated with a V_T_ >8 ml/kg^−1^. The correlation between baseline PPV values and the increase in CO induced by the fluid challenge was 0.23 (*P* <0.001) (Figure 2). Central venous pressure was available in 406 patients.

### Comparisons between responders and non-responders

An increase in SV ≥15% was observed in 267 patients (48%) defined as responders. The comparisons between responders and non-responders are shown in Table 1.

**Table 1:** **Characteristics of responders (increase in stroke volume ≥15% after volume expansion) and non-responders to fluid challenge**

	**Responders**	**Non-responders**	***P*** **value**
	**(n = 267)**	**(n = 289)**	
**Age**	58 ± 18	56 ± 17	0.40
**Sex (women/men)**	92/175	105/183	0.68
**Height (cm)**	169 ± 9 (n = 266)	169 ± 9 (n = 288)	0.72
**Weight (kg)**	72 ± 15 (n = 266)	75 ± 17 (n = 288)	0.053
**IBW (kg)**	63 ± 10 (n = 266)	63 ± 9 (n = 288)	0.76
**SAPS II score**	50 ± 20 (n = 147)	53 ± 20 (n = 188)	0.13
**HR (beats/min)**	91 ± 24	88 ± 25	0.066
**MAP(mmHg)**	73 ± 15	75 ± 16	0.12
**RR (cycle/min)**	19 ± 6 (n = 231)	19 ± 6 (n = 262)	0.55
**V**_**T**_ **/IBW (ml/kg)**	7.4 ± 2.3 (n = 248)	7.6 ± 2.6 (n = 271)	0.57
**V**_**T**_ **/IBW <8 ml/kg (%)**	109 (41%)	134 (46%)	0.24
**PEEP**	6 ± 5 (n = 249)	7 ± 5 (n = 271)	0.095
**PPlat (cmH**_**2**_ **O)**	19.3 ± 5.5 (n = 205)	20.7 ± 5.8 (n = 225)	0.014
**Driving pressure (cmH**_**2**_ **O)**	14 ± 5 (n = 192)	15 ± 5 (n = 211)	0.055
**C**_**st,rs**_ **(ml/cmH**_**2**_ **O)**	39.5 ± 14.3 (n = 192)	36.0 ± 13.5 (n = 211)	0.011
**HR/RR**	5.0 ± 1.5 (n = 231)	4.8 ± 1.6 (n = 262)	0.073
**HR/RR ≤3.6 (n,%)**	231 (17.8%)	262 (27.1%)	0.018
**PaO**_**2**_ **/FiO**_**2**_ **ratio**	185 ± 107 (n = 111)	182 ± 103 (n = 135)	0.85
**Norepinephrine infusion**	--	--	--
**Dosage (μ.kg** ^**−1**^ **/min** ^**−1**^ **)**	0.49 ± 0.67 (n = 158)	0.47 ± 0.63 (n = 172)	0.75
**CVP (mmHg)**	9 ± 5	11 ± 4	<0.001
**Baseline SV**	60 ± 21	74 ± 27	<0.001
**PPV (%)**	15 ± 9	9 ± 7	<0.001

Data are presented as means ± standard deviation (SD); n = number of available data. CVP, central venous pressure; C_st,rs_, static compliance of the respiratory system; driving pressure = plateau pressure - positive end-expiratory pressure; HR, heart rate; IBW, ideal body weight; MAP, mean arterial pressure; PaO_2_/FiO_2_ ratio, partial arterial oxygen pressure to inspiratory oxygen fraction ratio; PEEP, positive end-expiratory pressure; PPlat, plateau pressure; PPV: pulse pressure variation; RR, respiratory rate; SAPS II, Simplified Acute Physiology Score II; SV, stroke volume; V_T_, tidal volume.

### Grey zone approach for the overall population

**Figure 3:**
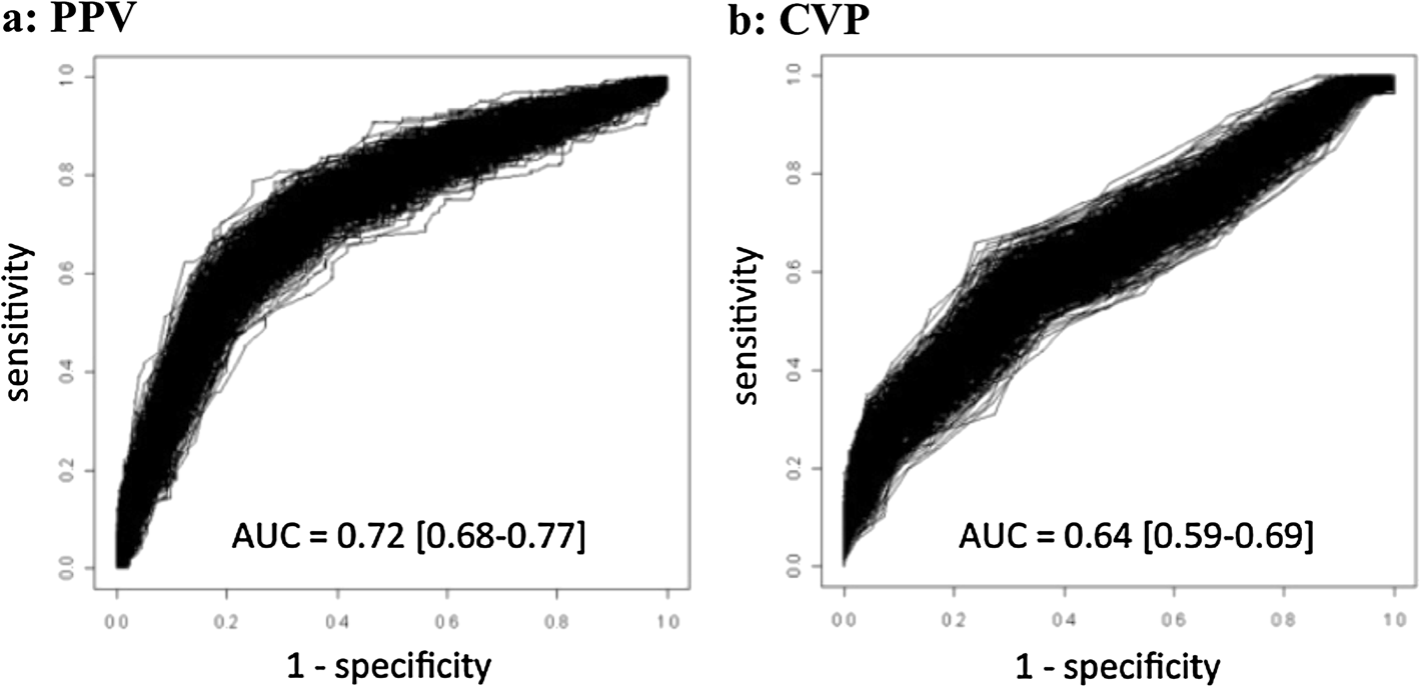
**Bootstrapping of ROC curves of pulse pressure variation (PPV) (a) and central venous pressure (CVP) (b).** ROC curve areas are expressed as mean value with 95% confidence interval.

Using a bootstrap analysis, the median values of the area under the ROC curve of PPV and CVP were 0.73 (95% CI = 0.68 to 0.77) and 0.64 (95% CI = 0.59 to 0.70), respectively (*P* <0.001) (Figure 3). The 95% CIs for the best threshold values were 9 to 14% and 6 to 9 mmHg, respectively. Because no unique best cut-off value can be obtained using the bootstrap method, the best cut-off values for PPV and CVP with the ROC curve were obtained from the original population (without bootstrap analysis). These best cut-off values were 7% (specificity = 0.71, sensitivity = 0.63, positive likelihood ratio = 2.17, negative likelihood ratio = 0.52) and 10 mmHg (specificity = 0.67, sensitivity = 0.70, positive likelihood ratio = 2.12, negative likelihood ratio = 0.45) for PPV and CVP, respectively. There were 96/564 (17%) patients with a PPV value <4%.

**Figure 4:**
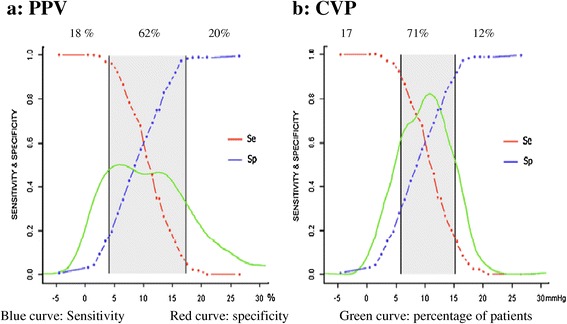
**Grey zones of pulse pressure variation (PPV) (a) and central venous pressure (CVP) (b).** Blue curve: sensitivity; red curve: specificity; green curve: percentage of patients.

Using the alternative grey zone approach, inconclusive values spreading from 4 to 17% and from 6 to 15 mmHg were found for PPV and CVP, respectively (Figure 4). There were 62% and 71% patients in the grey zone of PPV and CVP, respectively (*P* <0.01).

### Factors influencing the ability of PPV to predict fluid responsiveness

**Figure 5:**
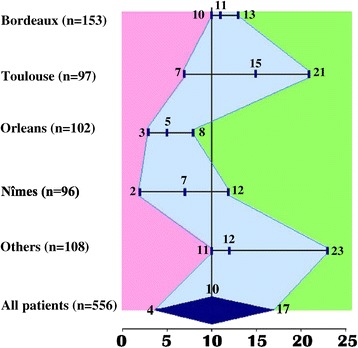
**Grey zone according to the centre (centres in which the number of inclusion was under 70 were pooled together) and factors influencing on the ability of PPV to predict fluid responsiveness.** Each bar represents the grey zone of the corresponding centre with its lowest limit (specificity ≥90%), its best threshold and its highest limit (sensitivity ≥90%).

Table 2 shows the influence of the following variables that have been shown to influence the ability of PPV to predict fluid responsiveness: V_T_, driving pressure (plateau pressure - PEEP), respiratory compliance (V_T_/(plateau pressure - PEEP), HR/RR ratio, use of vasopressors. Only a V_T_ ≥8 ml/kg^−1^ (*P* <0.001) and a driving pressure >20 cmH_2_O (*P* <0.001) were associated with a significantly greater area under the ROC curve for PPV. Figure 5 shows the grey zones according to the considered centre (Amiens, Lyon and Paris were studied as ‘others’ because the numbers of included patients were small), and factors that could influence the ability to discriminate responders and non-responders.

**Table 2:** **Ability of pulse pressure variations to predict an increase of more than 15% in cardiac output after volume expansion according to tidal volume, driving pressure, respiratory compliance, heart rate/respiratory rate ratio and vasopressor use**

	**AUC ROC 95% CI**	**Optimal threshold (%)**	**Gray zone**
**V**_**T**_ **/IBW (ml/kg)**			
**<8 (n = 280)**	0.69 (0.64-0.73)	9	2-17
≥**8 (n = 276)**	0.77 (0.73-0.81)^a^	12	8-19
**Driving pressure (cm H**_**2**_ **0)**			
≤ **20 (n = 356)**	0.67 (0.63-0.72)	7	3-17
**>20 (n = 51)**	0.78 (0.74-0.82)^b^	14	12-21
**C**_**st,rs**_ **(mL/cm H**_**2**_ **0)**			
≤ **30 (n = 145)**	0.60 (0.51-0.70)	12	2-21
**>30 (n = 262)**	0.73 (0.67-0.79)	7	3-17
**HR/RR**			
≤ **3.6 (n = 114)**	0.65 (0.54-0.76)	7	1-12
**>3.6 (n = 384)**	0.73 (0.68-0.78)	10	5-17
**Vasopressor**			
**Yes (n = 249)**	0.70 (0.66-0.75)	14	3-16
**No (n = 307)**	0.72 (0.69-0.77)	10	6-18

Data are presented as medians (95% confidence intervals). ^a^: *P* <0.001 vs <8 ml.kg^−1^; ^b^: *P* <0.001 vs ≤20 cm H_2_0). AUC, area under the receiver operating characteristics curves, C_st,rs,_ static compliance of the respiratory system; HR/RR, heart rate/respiratory rate ratio; V_T_ /IBW, tidal volume/ideal body weight ratio.

### Risk/benefit analysis

A P_a_O_2_/F_i_O_2_ ratio was available for 250 patients. For the severe group (n = 57), the best threshold value for PPV was 7%, AUC = 0.67 (95% CI = 0.54 to 0.80), grey zone = 1% to 12%; for the moderate group (n = 111), the best threshold value for PPV was 5%, AUC = 0.73 (95% CI = 0.60 to 0.80), grey zone = 2 to 14%; for the mild group (n = 82) the best PPV threshold value was 12%, AUC = 0.67 (95% CI = 0.57 to 0.74), grey zone = 2 to 14%. For the whole population, when the clinical risk of fluid infusion during ARDS was taken into account by the ratio of costs for severe and mild groups, the best threshold values for PPV and CVP were 14% and 2 mmHg, respectively. When taking into account the risk of fluid infusion in the whole population, the grey zone for PPV was 2% to 13%.

## Discussion

The present study demonstrated that, when using a grey zone approach in a large population of mixed ICU patients: (1) PPV cannot reliably predict fluid responsiveness when its value is between 4% to 17%; (2) 62% of patients (with criteria of valid PPV) had PPV values within this grey zone; (3) a V_T_ ≥8 ml/kg^−1^ and/or driving pressure >20 cmH_2_O significantly improve the ability of PPV to predict fluid responsiveness; and (4) even when taking into account the risk of fluid loading in ARDS patients, the grey zone remains too large (2 to 13%) to be informative.

Our results are in accordance with previous large reports that emphasized the superiority of dynamic over static variables, but contrast with the very high predictive value of PPV previously reported [1,43]. This apparent contradiction is mainly due to substantial changes in ventilatory practices during the past 15 years (the lung protective strategy) in the ICU. The lung protective strategy is based on the use of low V_T_ in patients with ARDS to prevent baro- or volo-trauma. In the early 2000s, dynamic variables were validated in critically ill patients ventilated with high V_T_ (>8 mL/kg^−1^ of IBW) [1,8,9,43,44]. Subsequent studies showed that low V_T_, low driving pressure or low pulmonary compliance significantly impairs the ability of dynamic variables to predict fluid responsiveness [10,11,13,15,45,46]. Because the present study involved ICU patients who were mainly (but not systematically) mechanically ventilated with low V_T_ (<8 ml/kg^−1^ of IBW), our hypothesis is that low V_T_ is the main explanation for the poor predictive performance of PPV. This hypothesis is supported by the fact that PPV accuracy is improved in patients with driving pressure >20 cmH_2_O and V_T_ >8 mL/kg^−1^. The PPV is due to the transmission of pulmonary pressure to the intra-thoracic circulatory compartment and thus, V_T_ and driving pressure directly participate in the pressure transmitted to intra-thoracic components. Surprisingly, in the present report, the compliance of the respiratory system did not modify the ability of PPV to predict fluid responsiveness. In a recent study, Monnet *et al*. [46] showed that the ability of PPV to predict fluid responsiveness was inversely related to compliance but not to V_T_. To elucidate such conflicting results, a study using oesophageal pressure (reflecting pressure in the pleural space) could be useful to precise the effect of pulmonary pressure on PPV [47]. Finally, the poor predictive performance of PPV reported in the present study may be due to the relatively low level of PEEP. Freitas *et al*. [48] recently reported that automated PPV accurately predicted fluid responsiveness in septic patients using a protective lung ventilation strategy (V_T_ =6 mL/kg^−1^). However, in this study, a relatively high PEEP level (>10 cmH_2_O) was applied in contrast with the present report and with Monnet’s [46] study (average PEEP =6 cmH_2_O). Indeed, a high level of PEEP could facilitate the transmission of pulmonary pressure to the intra-thoracic circulatory compartment and explain these apparent conflicting results. Specific studies are needed to elucidate this point especially with recording of pleural pressure.

In the present study, pooled data were obtained from studies where V_T_ >7 to 8 ml/kg was an inclusion criterion, with positive results (that is, a high predictive value for fluid responsiveness) and cut-off values around 12% [27,28,32] with studies performed in patients ventilated with smaller tidal volumes (around 6 ml/kg), where poor results and low cut-off values (around 5 to 7%) were reported [10,32]. This wide range of tidal volume can explain the importance of the grey zone value (4 to 17%) and the variation of grey zone value among centres. This value is considerably larger in those previously published in surgical patients ventilated with relatively high and fixed tidal volume (8 mL/kg) that ranged from 9 to 13% [18]. The present results show that, whatever tidal volume value, a PPV value <17% lead to a risk of false negative. The present results also show that a high PPV value (>17%) whatever the tidal volume value is often associated with a positive fluid responsiveness. Finally, in the present report, a V_T_ ≥8 ml/kg^−1^ and/or a driving pressure >20 cmH_2_O significantly improve the ability of PPV to predict fluid responsiveness.

The potential deleterious consequences of unwarranted fluid infusion are crucial during ARDS since a restrictive strategy has been shown to reduce mechanical ventilation duration in these patients [5,40,41]. The grey zone in the severe, moderate or mild ARDS groups was too large to be clinically informative. For severe ARDS patients (P_a_O_2_/F_i_O_2_ ratio <100), we considered that the risk of excessive fluid infusion was twice as high when compared with less severe patients. For the severe ARDS group, our results show that when applying a cost ratio of 2 (two-fold increased risk in case of fluid infusion) the best cut-off value for PPV moved from 7 to 14%. This suggests that the more severe the ARDS, the higher the cut-off, in order to limit the risk of unnecessary fluid loading. Nevertheless, because the grey zones were 1 to 12% for the moderate ARDS group, 1 to 14% for the mild ARDS group and 2 to 14% for the severe ARDS group, we failed to show any relevant difference in the grey zone regardless of severity.

Clinical implications: when a given value of PPV is in the grey zone, physicians cannot use this index to proceed with or to exclude the need for fluid therapy in the ICU. In such a case, a passive leg-raising test, an end-expiratory occlusion test or ‘minifluid’ challenge could be valuable alternatives in ventilated patients with low V_T_ <8 mL.kg^−1^ even if these indices have yet to be assessed with a grey zone approach [32,46]. Conversely, when a measured value is outside of the grey zone, the necessity of performing a fluid challenge can be confirmed (value above the upper limit of the grey zone) or excluded (value below the lower limit of the grey zone) with less than 10% error (specificity and sensitivity >90%). At the bedside, the grey zone appears to be more informative than the confidence interval of the best threshold value obtained with the ROC curve methodology [16,18,19]. Interestingly, the grey zone varied according to the considered centre. This probably reflected the different management of such patients in each centre. Therefore, this finding probably means that the concept of the grey zone should be adapted according to the local policy of fluid challenge, mechanical ventilation and others factors that could influence PPV predictive value.

The present study shows a grey zone of 4 to 17%, meaning that PPV cannot reliably guide fluid loading in 62% of studied population. Only rarely (17% of the study population), when under the threshold of 4%, does PPV rule out any use of fluid loading, which could then be avoided. In contrast, a PPV value above 17% can be considered as a useful tool to indicate fluid infusion. Nevertheless, an isolated value of PPV (even above 17%) cannot be considered as the sole argument to decide fluid therapy. The latter should be only considered when signs of a clinical hypoperfusion are associated with PPV value above 17%. In other words, PPV should be considered as a tool, not as a target. Moreover, the great variability according to the centres suggests that the patient case mix and different management protocols could influence the reliability of PPV to predict fluid responsiveness.

It could be hypothesized that the important variation of grey zone value among centres probably reflects the fact that PPV was measured by different techniques. In particular, PPV was manually or automatically measured according to each study centre’s procedures. This heterogeneity reflects the real daily practice of PPV. There may be differences between absolute PPV values obtained from automated or manual calculation. There may also be differences between different automatic devices. Finally, it is also possible that actual criteria for accurate PPV measurement were not uniform among centres at the time of study, especially for right ventricle failure detection [49]. This highlights the fact that, as previously demonstrated for filling pressure a rigorous technique for PPV measurement is of particular importance [50]. In the same way, it could be objected that the conditions of PPV validity chosen for the present report do not reflect the actual recommendation. The criteria used reflect what was recommended at the time of publication of the main studies used for the present report [49]. These criteria do not reflect the actual recommendations, especially for tidal volume and right ventricle failure [49].

Our study presents several limitations that should be considered when assessing the clinical relevance of our results. First, this study is not prospective, which limits the power of the conclusions. Second, the methods of PPV and CO measurements were not uniform and this may have extended the PPV grey zone. However, thermodilution and echocardiography have both been validated and this methodological issue was considered as acceptable in a previous report [18]. Furthermore, patients with clinical or suspected intra-abdominal pressure syndrome were excluded because the latter can affect dynamic indicators of fluid responsiveness, especially by increasing the threshold value of PPV [51,52]. However, a clinical examination (as performed in the present study) cannot rule out an intra-abdominal pressure syndrome. The lack of direct measurement of intra-abdominal pressure could partially explain the large grey zone found in the present study [52]. Third, the present study pooled the findings of different centres and different ICU populations. Figure 5 clearly shows the heterogeneity between centres, corresponding to the real PPV daily practice. Fourth, the present main finding, that is a grey zone of PPV between 4 to 17%, should be validated in a prospective study including different patients in different ICUs. Fifth, the existence of a right ventricle failure was not systematically ruled out before measuring PPV in hypoxemic patients. This is due to the fact that, at the time of publication of the main studies involved in the present report, right ventricle failure was not a usual non-validity criterion for PPV assessment. Sixth, as the baseline levels of stroke volume are substantially different in responders and non-responders, we cannot rule out the fact that our findings reflect regression artefact. Finally, as mentioned above, the relation between PPV accuracy, respiratory system compliance, driving pressure or PEEP level remains unclear and is still debated. Physiological studies measuring pleural space pressure or oesophageal pressure may better explain the decreased reliability of PPV for predicting fluid responsiveness in mechanically ventilated ICU patients with low V_T_. All these limitations are emphasized by the limited number of patients in whom PPV can be measured. Indeed, recommendations in sedation and in mechanically ventilation tend to favour spontaneously breathing modes in order to decrease the duration of mechanical ventilation [53,54]. These practices may decrease the clinical utility of PPV as recently reported in the anaesthetic setting [55]. In addition, it must be kept in mind that the increase in stroke volume is not always associated to a greater oxygen delivery to cells that is the main objective of a fluid challenge.

## Conclusions

In ventilated ICU patients, the grey zone approach identifies a wide range of PPV values, between 4 and 17%, for which fluid responsiveness cannot be accurately predicted, corresponding to 62% of patients in whom criteria for measuring PPV are valid. The heterogeneity in measurement method and in tidal volume value can contribute to the present findings.

## Key messages

- In 564 ICU mechanically ventilated patients, a grey zone approach showed that pulsed pressure variations (PPV) cannot reliably predict fluid responsiveness when its value is between 4% and 17%
- In this population, 62% of patients (with criteria of valid PPV) had PPV values within this grey zone
- A V_T_ ≥8 ml/kg^−1^ and/or driving pressure >20 cmH_2_O significantly improve the ability of PPV to predict fluid responsiveness
- Even when taking into account the risk of fluid loading in ARDS patients, the grey zone remains too large (2 to 13%) to be informative.

## Abbreviations

ARDS: acute respiratory distress syndrome
BMI: body mass index
CI: cardiac index
CI: confident interval
CO: cardiac output
CVP: central venous pressure
F_i_O_2_: inspiratory oxygen fraction
HR: heart rate
IBW: ideal body weight
ICU: intensive care unit
MAP: mean arterial pressure
NR: non-responder
P_a_O_2_: partial arterial oxygen pressure
PEEP: positive end-expiratory pressure
PPmax: maximum value of pulse pressure
PPmin: minimum value of pulse pressure
PPV: pulse pressure variation
R: responder
ROC: receiver operating curve
RR: respiratory rate
SD: standard deviation
SV: stroke volume
V_T_: tidal volume

## References

[1] Michard F, Teboul JL (2002). Predicting fluid responsiveness in ICU patients: a critical analysis of the evidence. Chest.

[2] Jansen TC, van Bommel J, Schoonderbeek FJ, Sleeswijk Visser SJ, van der Klooster JM, Lima AP, Willemsen SP, Bakker J, group Ls (2010). Early lactate-guided therapy in intensive care unit patients: a multicenter, open-label, randomized controlled trial. Am J Respir Crit Care Med.

[3] Jones AE, Shapiro NI, Trzeciak S, Arnold RC, Claremont HA, Kline JA, Emergency Medicine Shock Research Network I (2010). Lactate clearance vs central venous oxygen saturation as goals of early sepsis therapy: a randomized clinical trial. JAMA.

[4] Boyd JH, Forbes J, Nakada TA, Walley KR, Russell JA (2011). Fluid resuscitation in septic shock: a positive fluid balance and elevated central venous pressure are associated with increased mortality. Crit Care Med.

[5] National Heart L, Wiedemann HP, Wheeler AP, Bernard GR, Thompson BT, Hayden D, deBoisblanc B, Connors AF, Hite RD, Harabin AL, Blood Institute Acute Respiratory Distress Syndrome Clinical Trials N (2006). Comparison of two fluid-management strategies in acute lung injury. N Engl J Med.

[6] Payen D, de Pont AC, Sakr Y, Spies C, Reinhart K, Vincent JL, Sepsis Occurrence in Acutely Ill Patients I (2008). A positive fluid balance is associated with a worse outcome in patients with acute renal failure. Crit Care.

[7] Bouchard J, Soroko SB, Chertow GM, Himmelfarb J, Ikizler TA, Paganini EP, Mehta RL, Program to Improve Care in Acute Renal Disease Study G (2009). Fluid accumulation, survival and recovery of kidney function in critically ill patients with acute kidney injury. Kidney Int.

[8] Michard F, Boussat S, Chemla D, Anguel N, Mercat A, Lecarpentier Y, Richard C, Pinsky MR, Teboul JL (2000). Relation between respiratory changes in arterial pulse pressure and fluid responsiveness in septic patients with acute circulatory failure. Am J Respir Crit Care Med.

[9] Michard F (2005). Changes in arterial pressure during mechanical ventilation. Anesthesiology.

[10] Muller L, Louart G, Bousquet PJ, Candela D, Zoric L, de La Coussaye JE, Jaber S, Lefrant JY (2010). The influence of the airway driving pressure on pulsed pressure variation as a predictor of fluid responsiveness. Intensive Care Med.

[11] De Backer D, Heenen S, Piagnerelli M, Koch M, Vincent JL (2005). Pulse pressure variations to predict fluid responsiveness: influence of tidal volume. Intensive Care Med.

[12] De Backer D, Taccone FS, Holsten R, Ibrahimi F, Vincent JL (2009). Influence of respiratory rate on stroke volume variation in mechanically ventilated patients. Anesthesiology.

[13] Vallee F, Richard JC, Mari A, Gallas T, Arsac E, Verlaan PS, Chousterman B, Samii K, Genestal M, Fourcade O (2009). Pulse pressure variations adjusted by alveolar driving pressure to assess fluid responsiveness. Intensive Care Med.

[14] Huang CC, Fu JY, Hu HC, Kao KC, Chen NH, Hsieh MJ, Tsai YH (2008). Prediction of fluid responsiveness in acute respiratory distress syndrome patients ventilated with low tidal volume and high positive end-expiratory pressure. Crit Care Med.

[15] Lakhal K, Ehrmann S, Benzekri-Lefevre D, Runge I, Legras A, Dequin PF, Mercier E, Wolff M, Regnier B, Boulain T (2011). Respiratory pulse pressure variation fails to predict fluid responsiveness in acute respiratory distress syndrome. Crit Care.

[16] Coste J, Jourdain P, Pouchot J (2006). A gray zone assigned to inconclusive results of quantitative diagnostic tests: Application to the use of brain natriuretic peptide for diagnosis of heart failure in acute dyspneic patients. Clin Chem.

[17] Ray P, Le Manach Y, Riou B, Houle TT (2010). Statistical evaluation of a biomarker. Anesthesiology.

[18] Cannesson M, Le Manach Y, Hofer CK, Goarin JP, Lehot JJ, Vallet B, Tavernier B (2011). Assessing the diagnostic accuracy of pulse pressure variations for the prediction of fluid responsiveness: a “gray zone” approach. Anesthesiology.

[19] Coste J, Pouchot J (2003). A grey zone for quantitative diagnostic and screening tests. Int J Epidemiol.

[20] Feinstein AR, Cicchetti DV (1990). High agreement but low kappa: I. The problems of two paradoxes. J Clin Epidemiol.

[21] Jaber S, Coisel Y, Chanques G, Futier E, Constantin JM, Michelet P, Beaussier M, Lefrant JY, Allaouchiche B, Capdevila X, Marret E (2012). A multicentre observational study of intra-operative ventilatory management during general anaesthesia: tidal volumes and relation to body weight. Anaesthesia.

[22] Herasevich V, Tsapenko M, Kojicic M, Ahmed A, Kashyap R, Venkata C, Shahjehan K, Thakur SJ, Pickering BW, Zhang J, Hubmayr RD, Gajic O (2011). Limiting ventilator-induced lung injury through individual electronic medical record surveillance. Crit Care Med.

[23] Lellouche F, Lipes J (2013). Prophylactic protective ventilation: lower tidal volumes for all critically ill patients?. Intensive Care Med.

[24] Boutin C, Cohendy R, Muller L, Jaber S, Mercat A, Brochard L, Richard JC, Fabbro-Peray P, Ripart J, de La Coussaye JE, Lefrant JY (2010). Impact of express study on clinical practice in ARDS patients: a single French ICU experience. Ann Fr Anesth Reanim.

[25] Determann RM, Royakkers A, Wolthuis EK, Vlaar AP, Choi G, Paulus F, Hofstra JJ, de Graaff MJ, Korevaar JC, Schultz MJ (2010). Ventilation with lower tidal volumes as compared with conventional tidal volumes for patients without acute lung injury: a preventive randomized controlled trial. Crit Care.

[26] Futier E, Constantin JM, Paugam-Burtz C, Pascal J, Eurin M, Neuschwander A, Marret E, Beaussier M, Gutton C, Lefrant JY, Allaouchiche B, Verzilli D, Leone M, De Jong A, Bazin JE, Pereira B, Jaber S, IMPROVE Study Group (2013). A trial of intraoperative low-tidal-volume ventilation in abdominal surgery. N Engl J Med.

[27] Lakhal K, Ehrmann S, Runge I, Benzekri-Lefevre D, Legras A, Dequin PF, Mercier E, Wolff M, Regnier B, Boulain T (2010). Central venous pressure measurements improve the accuracy of leg raising-induced change in pulse pressure to predict fluid responsiveness. Intensive Care Med.

[28] Biais M, Nouette-Gaulain K, Cottenceau V, Revel P, Sztark F (2008). Uncalibrated pulse contour-derived stroke volume variation predicts fluid responsiveness in mechanically ventilated patients undergoing liver transplantation. Br J Anaesth.

[29] Biais M, Cottenceau V, Stecken L, Jean M, Ottolenghi L, Roullet S, Quinart A, Sztark F (2012). Evaluation of stroke volume variations obtained with the pressure recording analytic method. Crit Care Med.

[30] Mahjoub Y, Benoit-Fallet H, Airapetian N, Lorne E, Levrard M, Seydi AA, Amennouche N, Slama M, Dupont H (2012). Improvement of left ventricular relaxation as assessed by tissue Doppler imaging in fluid-responsive critically ill septic patients. Intensive Care Med.

[31] Pottecher J, Deruddre S, Teboul JL, Georger JF, Laplace C, Benhamou D, Vicaut E, Duranteau J (2010). Both passive leg raising and intravascular volume expansion improve sublingual microcirculatory perfusion in severe sepsis and septic shock patients. Intensive Care Med.

[32] Muller L, Toumi M, Bousquet PJ, Riu-Poulenc B, Louart G, Candela D, Zoric L, Suehs C, de La Coussaye JE, Molinari N, Lefrant JY, AzuRéa Group (2011). An increase in aortic blood flow after an infusion of 100 ml colloid over 1 minute can predict fluid responsiveness: the mini-fluid challenge study. Anesthesiology.

[33] Knaus WA, Draper EA, Wagner DP, Zimmerman JE (1985). APACHE II: a severity of disease classification system. Crit Care Med.

[34] Le Gall JR, Lemeshow S, Saulnier F (1993). A new Simplified Acute Physiology Score (SAPS II) based on a European/North American multicenter study. JAMA.

[35] Reuter DA, Felbinger TW, Schmidt C, Kilger E, Goedje O, Lamm P, Goetz AE (2002). Stroke volume variations for assessment of cardiac responsiveness to volume loading in mechanically ventilated patients after cardiac surgery. Intensive Care Med.

[36] Carpenter J, Bithell J (2000). Bootstrap confidence intervals: when, which, what? A practical guide for medical statisticians. Stat Med.

[37] DeLong ER, DeLong DM, Clarke-Pearson DL (1988). Comparing the areas under two or more correlated receiver operating characteristic curves: a nonparametric approach. Biometrics.

[38] Le Manach Y, Hofer CK, Lehot JJ, Vallet B, Goarin JP, Tavernier B, Cannesson M (2012). Can changes in arterial pressure be used to detect changes in cardiac output during volume expansion in the perioperative period?. Anesthesiology.

[39] Wiedemann HP, Wheeler AP, Bernard GR, Thompson BT, Hayden D, deBoisblanc B, Connors AF, Hite RD, Harabin AL (2006). Comparison of two fluid-management strategies in acute lung injury. N Engl J Med.

[40] Sakr Y, Vincent JL, Reinhart K, Groeneveld J, Michalopoulos A, Sprung CL, Artigas A, Ranieri VM (2005). High tidal volume and positive fluid balance are associated with worse outcome in acute lung injury. Chest.

[41] Murphy CV, Schramm GE, Doherty JA, Reichley RM, Gajic O, Afessa B, Micek ST, Kollef MH (2009). The importance of fluid management in acute lung injury secondary to septic shock. Chest.

[42] Ranieri VM, Rubenfeld GD, Thompson BT, Ferguson ND, Caldwell E, Fan E, Camporota L, Slutsky AS (2012). Acute respiratory distress syndrome: the Berlin Definition. JAMA.

[43] Marik PE, Cavallazzi R, Vasu T, Hirani A (2009). Dynamic changes in arterial waveform derived variables and fluid responsiveness in mechanically ventilated patients: a systematic review of the literature. Crit Care Med.

[44] Bendjelid K, Romand JA (2003). Fluid responsiveness in mechanically ventilated patients: a review of indices used in intensive care. Intensive Care Med.

[45] Renner J, Cavus E, Meybohm P, Gruenewald M, Steinfath M, Scholz J, Boening A, Bein B (2008). Pulse pressure variation and stroke volume variation during different loading conditions in a paediatric animal model. Acta Anaesthesiol Scand.

[46] Monnet X, Bleibtreu A, Ferre A, Dres M, Gharbi R, Richard C, Teboul JL (2012). Passive leg-raising and end-expiratory occlusion tests perform better than pulse pressure variation in patients with low respiratory system compliance. Crit Care Med.

[47] Lakhal K, Ehrmann S, Boulain T (2012). Pulse pressure variation: does lung compliance really matter?. Crit Care Med.

[48] Freitas FG, Bafi AT, Nascente AP, Assuncao M, Mazza B, Azevedo LC, Machado FR (2013). Predictive value of pulse pressure variation for fluid responsiveness in septic patients using lung-protective ventilation strategies. Br J Anaesth.

[49] Biais M, Ouattara A, Janvier G, Sztark F (2012). Case scenario: respiratory variations in arterial pressure for guiding fluid management in mechanically ventilated patients. Anesthesiology.

[50] Gnaegi A, Feihl F, Perret C (1997). Intensive care physicians’ insufficient knowledge of right-heart catheterization at the bedside: time to act?. Crit Care Med.

[51] Duperret S, Lhuillier F, Piriou V, Vivier E, Metton O, Branche P, Annat G, Bendjelid K, Viale JP (2007). Increased intra-abdominal pressure affects respiratory variations in arterial pressure in normovolaemic and hypovolaemic mechanically ventilated healthy pigs. Intensive Care Med.

[52] Kirkpatrick AW, Roberts DJ, De Waele J, Jaeschke R, Malbrain ML, De Keulenaer B, Duchesne J, Bjorck M, Leppaniemi A, Ejike JC, Sugrue M, Cheatham M, Ivatury R, Ball CG, Reintam Blaser A, Regli A, Balogh ZJ, D'Amours S, Debergh D, Kaplan M, Kimball E, Olvera C, Pediatric Guidelines Sub-Committee for the World Society of the Abdominal Compartment Syndrome (2013). Intra-abdominal hypertension and the abdominal compartment syndrome: updated consensus definitions and clinical practice guidelines from the World Society of the Abdominal Compartment Syndrome. Intensive Care Med.

[53] Barr J, Fraser GL, Puntillo K, Ely EW, Gelinas C, Dasta JF, Davidson JE, Devlin JW, Kress JP, Joffe AM, Coursin DB, Herr DL, Tung A, Robinson BR, Fontaine DK, Ramsay MA, Riker RR, Sessler CN, Pun B, Skrobik Y, Jaeschke R, American College of Critical Care Medicine (2013). Clinical practice guidelines for the management of pain, agitation, and delirium in adult patients in the intensive care unit. Crit Care Med.

[54] Chanques G, Kress JP, Pohlman A, Patel S, Poston J, Jaber S, Hall JB (2013). Impact of ventilator adjustment and sedation-analgesia practices on severe asynchrony in patients ventilated in assist-control mode. Crit Care Med.

[55] Maguire S, Rinehart J, Vakharia S, Cannesson M (2011). Technical communication: respiratory variation in pulse pressure and plethysmographic waveforms: intraoperative applicability in a North American academic center. Anesth Analg.

